# The complete chloroplast genome of *Polygonatum odoratum* (Liliaceae), an endemic medicinal herb

**DOI:** 10.1080/23802359.2020.1806752

**Published:** 2020-11-06

**Authors:** Ze-Huan Wang, Ya-Qiong Li

**Affiliations:** Faculty of Traditional Chinese Pharmacy, Yunnan University of Traditional Chinese Medicine, Kunming, Yunnan, People’s Republic of China

**Keywords:** Complete chloroplast genome, *Polygonatum odoratum*, phylogenetic analysis, Liliaceae

## Abstract

In this study, we sequenced the complete chloroplast genome of *Polygonatum odoratum* with Illumina sequencing technology. The complete chloroplast genome length is 156,082bp, shows a typical tetrad structure, which manifests as one large and one small single copy (LSC and SSC) regions of 85,009 and 18,513bp, isolated by two inverted repeat regions (IRs) of 26,280bp. This study annotated altogether 131 unique genes, consisting of 86 protein-encoding genes, 8 rRNA and 38tRNA. According to the maximum likelihood phylogenetic tree based on 8 complete chloroplast genomes, *P. odoratum* shows close association with additional *Maianthemum* genus. The chloroplast genome-wide for *P. odoratum* would help to conserving the precious natural populations.

*Polygonatum odoratum* (Mill.) Druce, a typical representative of the Liliaceae family, is a perennial herbaceous plant that is widely distributed in East Asia and Europe (Zhao et al. 2018). It has been found to contain several components with bioactive effects, including polysaccharides, steroidal glycosides, dipeptides, flavonoids, amino acids, and trace mineral elements(Lin et al. 1994; Deng et al. 2012). Its rhizomes are regarded as the medicinal parts of the plant, and have been used extensively to treat diseases such as rheumatic heart disease, hypoimmunity, cardiovascular diseases, and diabetes (China Pharmacopoeia Committee 2015). *Polygonatum odoratum* has been recognized as the endemic medicinal perennial herb because resources of this herb are diminishing due to uncontrolled harvesting. Therefore, it is necessary for us to learn more about its genetic data and pay more attention to it. Notably, the chloroplast genome-wide for *P. odoratum* would help to conserve the precious natural populations.

In this study, silica-gel-dried leaves of *P. odoratum* were collected from Xianggelila of Yunnan Province, China (99°39.844E, 27°53.684 N), and voucher specimens (PO201909001) were deposited in the Herbarium of Yunnan University of Chinese Medicine. Total genomic DNA was extracted with the CTAB method (Doyle and Doyle 1987). We sequenced the complete chloroplast genome with Illumina Hiseq X-Ten (Illumina, San Diego, CA, USA), and 2.16 GB of sequence data was generated.The reads of the complete chloroplast genome were assembled using *de novo* assembling constructed in SPAdes 3.9.1 (Bankevich et al. 2012) using kmer lengths of 21–105bp, followed by reference guided assembling conducted with Bandage 0.8.1 (Wick et al. 2015) and Geneious 9.1.4 (Kearse et al. 2012). *Polygonatum humile* (MN218691) was used as reference for annotation using GeSeq (Tillich et al. 2017), coupled with manual correction for boundaries. The circular chloroplast genome map was drawn usingthe OGDRAW program (Greiner et al. 2019). To identify the phylogenetic position of *P. odoratum*, the maximum likelihood (ML) tree was reconstructed based on 8 species complete chloroplast genomes by MEGA X (Kumar et al. 2018).

The complete chloroplast genome of *P. odoratum* was 156,082 bp in length (GenBank accession number: MT646047), the GC content was 37.7%. LSC and SSC contained 85,009 bp and 18,513bp, respectively, while IR was 26,280 bp in length. A total of 131 unique genes were annotated, including 38tRNA, 8 rRNA, and 85 protein-coding genes. Seven protein-coding genes, nine tRNA and four rRNA genes were duplicated in the IR regions. In total, 18 intron-containing gene were in the chloroplast genome of *P. odoratum* of which three genes (rps12, clpP and ycf3) include two introns and the rest include a single intron.

Six chloroplast genomes of *Polygonatum* and one outgroup chloroplast genome were used for constructing maximum likelihoob (bootstrap repeat is 1000). Phylogenetic trees show that *P. odoratum* and other *Polygonatum* species formed a monophyletic clade with 100% bootstrap support value (Figure 1). Alignment was conducted using MAFFT (Katoh and Standley 2013).The complete chloroplast genome of *P. odoratum* would help to understanding the genetic information and conserving the precious natural populations.

**Figure 1.**
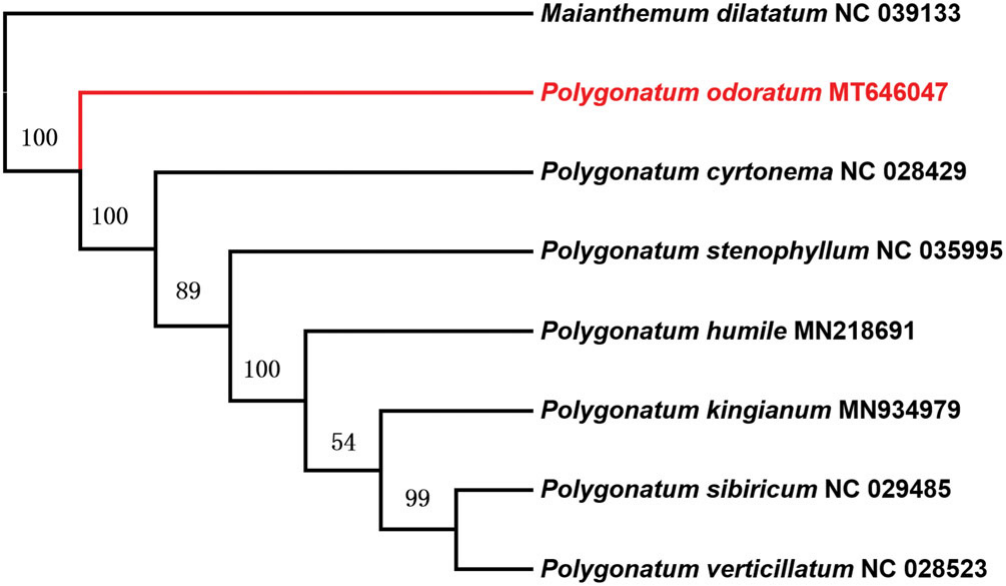
Maximum likelihood phylogenetic tree based on 8 complete chloroplast genomes (bootstrap repeat is 1000).

## Data Availability

The data that support the findings of this study are openly available in GenBank of NCBI at https://www.ncbi.nlm.nih.gov, reference number MT646047.
